# RW-BP100-4D, a Promising Antimicrobial Candidate With Broad-Spectrum Bactericidal Activity

**DOI:** 10.3389/fmicb.2021.815980

**Published:** 2022-01-25

**Authors:** Xingqi Tong, Jun Li, Ruicheng Wei, Lan Gong, Xing Ji, Tao He, Ran Wang

**Affiliations:** ^1^School of Food and Biological Engineering, Jiangsu University, Zhenjiang, China; ^2^Jiangsu Key Laboratory for Food Quality and Safety-State Key Laboratory Cultivation Base of Ministry of Science and Technology, Institute of Food Safety and Nutrition, Jiangsu Academy of Agricultural Sciences, Nanjing, China

**Keywords:** antimicrobial peptide, anti-biofilm, intracellular antibacterial activity, food disinfection, treatment efficacy

## Abstract

With the rapid emergence and dissemination of antimicrobial resistance (AMR) genes in bacteria from animal, animal-derived food and human clinic, it is of great significance to develop new approaches to combat the multidrug-resistant bacteria. This study presented a short linear antimicrobial peptide RW-BP100-4D, which was derived from RW-BP100 (RRLFRRILRWL-NH2) by transforming the N-terminal 4th amino acid from L- to D-enantiomer. This modification remarkably reduced the peptide cytotoxicity to mammalian cells, as indicated by hemolytic and cytotoxicity assays. Meanwhile, the antimicrobial activity of RW-BP100-4D was improved against a more variety of Gram-positive and Gram-negative bacteria (sensitive and resistant) as well as fungi. Also, RW-BP100-4D showed strong *in vitro* anti-biofilm activity in a concentration-dependent manner, including inhibition of the biofilm-formation and dispersion of the mature biofilms of *Staphylococcus aureus*. RW-BP100-4D could be efficiently uptaken by bovine mammary epithelial cells (MAC-T) cells to eliminate the intracellular *S. aureus* ATCC29213 and *Salmonella enterica* ATCC13076. Moreover, RW-BP100-4D was highly effective in food disinfection of multiple bacterial contamination (including *S. aureus*, *Listeria monocytogenesis*, *Escherichia coli* O157: H7, *Campylobacter jejuni*, *S. enterica*, and *Shewanella putrefaction*, 3.61 ± 0.063 log reduction) on chicken meat, and could kill 99.99% of the methicillin-resistant *Staphylococcus aureus* (MRSA) strain in the mouse skin infection model. In summary, RW-BP100-4D is a promising antimicrobial candidate for application on food disinfection and local infection treatment. However, the protease-sensitivity of RW-BP100-4D and toxic effect at higher doses reduced the therapeutic effect of the candidate peptide *in vivo* and should be improved in the future studies.

## Introduction

Antimicrobial peptides (AMPs) are the vital components of innate immune systems found in a wide range of organisms including plants, insects, amphibians, and humans (Zasloff, 2002; Mangoni and Shai, 2011; Pasupuleti et al., 2012). There are many types of AMPs including small bacteriocins, fungal defensins, peptaibols, cyclopeptides, and pseudopeptide (Raaijmakers et al., 2006; Montesinos, 2007; Mangoni and Shai, 2009), which can be used to treat microbial infections such as bacteria, viruses and fungi. Nowadays, with the emergence and prevalence of various antimicrobial resistance (AMR) genes in bacteria, AMPs are considered to be one of the most promising alternatives to conventional antibiotics (Mangoni and Shai, 2011). In general, most AMPs have amphiphilic structures with net positive charges (Zamora-Carreras et al., 2016). Due to the negative charge of the microbial cell membrane, the cationic AMPs could be easily accumulated on the cell membrane by an electrostatic affinity, thus leading to membrane lysis and cell content release (Toke, 2005; Bechinger and Gorr, 2017). Besides, other evidences have suggested that other antimicrobial mechanisms may be involved, such as targeting the intracellular substances (DNA or protein) and related biological process or pathways (Toke, 2005). Considering the complexity of the antimicrobial mechanisms of AMPs, it is believed that resistance to AMPs could hardly be developed (Brogden, 2005). In addition, due to the differences of net charge of anionic lipids between bacteria and eukaryotic cells, AMPs can be rationally designed to selectively target microorganisms, with low toxicity toward mammalian cells (Yeaman and Yount, 2003). This fact further supports the application of AMP as a novel intervention for the treatment of clinical infections *in vivo*, either alone or act synergistically with conventional antibiotics (Li et al., 2021).

BP100 is a short cationic AMP of 11 amino acids (KKLFKKILKYL-NH2), which is a hybrid of cecropin A (an AMP from the moth *Hyalophora cecropia*) and melittin M (a membrane-permeabilizing component of bee venom) (Ferre et al., 2009). This peptide displayed good antimicrobial activity against Gram-negative bacteria, with the minimal inhibitory concentration (MIC) of 2.5-5 μM against *Escherichia coli* and *Psudomnas aeruginosa* isolates. However, the antimicrobial activity of BP100 against Gram-positive bacteria was poor compared to that of Gram-negative bacteria. Subsequently, several BP100 analogues were designed and one derivative designated RW-BP100 (RRLFRRILRWL-NH2) showed expanded antibacterial activity against both Gram-negative and Gram-positive bacteria, whose Tyr residue was replaced with a Trp and all the Lys residues were substituted with Arg (Torcato et al., 2013). However, the cytotoxicity of RW-BP100 was not desirable. For instance, the hemolytic rate of RW-BP100 (4.9 μM) toward human erythrocytes was 50%, and the cell death rate of HeLa was 73% at the peptide concentration of 11 μM. Therefore, RW-BP100 may not be appropriate for further therapeutical application (Torcato et al., 2013).

To reduce the toxicity of RW-BP100 and improve its antimicrobial activity, a series of RW-BP100 derivatives were designed in this study, including L- to D-enantiomer at each amino acid position from 1st to 11th and the head-to-tail cyclized RW-BP100. According to the initial evaluation by cytotoxicity and antimicrobial activity tests, RW-BP100-4D (RRL{D-F}RRILRWL-NH2) was identified as the ideal candidate. Subsequently, RW-BP100-4D was further comprehensively evaluated for its stress (thermal, pH, and protease) stability, intracellular antibacterial activity, biofilm inhibition ability, food disinfection capacity, and treatment efficacy in mouse local and system infections.

## Materials and Methods

### Peptide Synthesis

**FIGURE 1:**
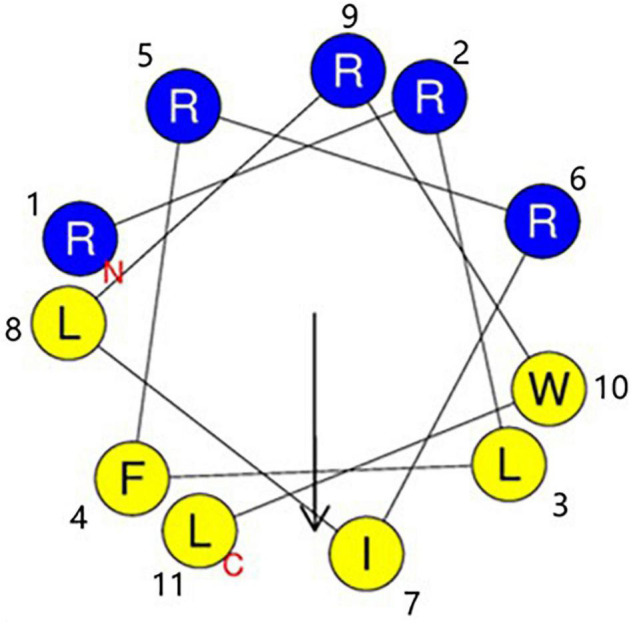
Edmunson wheel projection of the cationic peptide RW-BP100-4D. The structure was predicted at https://heliquest.ipmc.cnrs.fr/cgi-bin/ComputParams.py. RW-BP100-4D displays an amphiphilic structure with a net charge of 5. Blue background stands for hydrophilic amino acids, and yellow background for hydrophobic amino acids. N indicates the 1st amino acid (Arg) and T indicates the 11th amino acid (Leu).

The sequences of RW-BP100 and its serial derivatives were listed in Table 1, including the single substitution of L-amino acid with corresponding D-isomer at each position (RW-BP100-1∼11D), all D-isomers (RW-BP100-All-D), and the N to C-terminal cyclic RW-BP100 (RW-BP100-cycle). All the peptides were synthesized by GenScript Biotechnology Company (Nanjing, China) using the solid-phase method. The cationic peptides were of >95% purity and their structures were verified by mass spectrometry. The peptide powders were stored at −20°C until further experiment. The structural model of RW-BP100-4D was predicted at https://heliquest.ipmc.cnrs.fr/cgi-bin/ComputParams.py, and illustrated in Figure 1.

**TABLE 1:** Antibacterial activity of RW-BP100 and its derivatives.

Peptide	Sequence^a^	MIC (μg/mL)		Hemolytic rate (%)				Cell survival rate (%)^b^	
		*Staphylococcus aureus* ATCC29213	*Escherichia coli* ATCC25922	50 μg/mL	100 μg/mL	150 μg/mL	300 μg/mL	MAC-T	RAW264.7
RW-BP-100	RRLFRRILRWL	12	12	3.10 ± 0.77	4.70 ± 0.49	41.82 ± 2.27	56.67 ± 6.33	57.80 ± 3.67	36.29 ± 1.56
RW-BP100-1D	***R***RLFRRILRWL	25	12	4.61 ± 0.47	5.49 ± 0.23	39.81 ± 0.20	52.88 ± 3.06	52.71 ± 5.68	9.55 ± 2.45
RW-BP100-2D	R***R***LFRRILRWL	25	12	4.83 ± 1.08	5.99 ± 0.79	39.16 ± 1.75	62.12 ± 6.27	63.07 ± 3.23	6.83 ± 5.69
RW-BP100-3D	RR***L***FRRILRWL	25	50	3.49 ± 0.76	4.04 ± 0.20	38.13 ± 3.12	55.73 ± 5.27	50.63 ± 2.36	7.69 ± 7.89
RW-BP100-4D*	RRL***F***RRILRWL	3	6	0.11 ± 0.19	3.69 ± 0.01	18.75 ± 0.42	48.55 ± 2.53	92.68 ± 3.65	78.02 ± 7.66
RW-BP100-5D	RRLF***R***RILRWL	6	6	1.26 ± 0.54	2.49 ± 0.72	29.72 ± 0.94	51.41 ± 4.64	77.84 ± 5.63	39.05 ± 1.65
RW-BP100-6D	RRLFR***R***ILRWL	12	12	2.33 ± 1.51	3.25 ± 0.23	32.57 ± 1.20	55.81 ± 2.87	58.87 ± 3.45	5.92 ± 2.63
RW-BP100-7D	RRLFRR***I***LRWL	25	25	0.53 ± 1.12	1.11 ± 0.19	16.17 ± 3.36	45.65 ± 5.13	96.41 ± 3.75	93.74 ± 1.25
RW-BP100-8D	RRLFRRI***L***RWL	12	12	0.91 ± 0.38	1.38 ± 0.58	26.19 ± 0.10	50.11 ± 3.17	54.78 ± 2.65	20.30 ± 3.26
RW-BP100-9D	RRLFRRIL***R***WL	25	25	0.63 ± 0.24	1.53 ± 0.09	18.14 ± 0.20	45.18 ± 2.41	92.50 ± 2.36	91.33 ± 5.63
RW-BP100-10D	RRLFRRILR***W***L	50	50	0.31 ± 0.37	1.28 ± 0.19	19.20 ± 2.16	45.08 ± 4.70	94.41 ± 4.26	92.19 ± 6.32
RW-BP100-11D	RRLFRRILRW***L***	>50	>50	0.65 ± 0.60	1.42 ± 0.15	17.36 ± 1.92	46.97 ± 6.45	77.81 ± 5.28	83.27 ± 4.56
RW-BP100-All-D	***RRLFRRILRWL***	6	6	1.94 ± 0.94	2.75 ± 0.23	30.39 ± 2.11	51.40 ± 1.94	14.23 ± 2.37	23.37 ± 1.58
RW-BP100-cycle	RRLFRRILRWL	25	25	0.47 ± 0.61	1.19 ± 0.05	17.23 ± 3.08	48.67 ± 2.17	81.06 ± 2.16	44.23 ± 3.25

*^a^Amino acids in italic bold are D-enantiomers. RW-BP100-cycle indicates head-to-tail cyclized peptide. All peptides are C-terminal amides.*

*^b^Cell survival rate (%) was detected under the peptide concentration of 150 μg/mL.*

*Represents the peptide chosen as the ideal candidate for further analysis.

### Microbial Strains, Cell Lines, and Animals

Multiple bacterial species were used in this study. Standard strains included *Staphylococcus aureus* ATCC29213 and *S. aureus* ATCC33591 (*mecA*^+^), *Enterococcus faecalis* JH2-2, *Streptococcus agalactiae* ATCC13813, *Staphylococcus epidermidis* ATCC29887, *Listeria monocytogenes* ATCC19115, *E. coli* ATCC25922, *Salmonella enterica* ATCC13076, *Campylobacter jejuni* ATCC 33291, *Shewanella putrefaction* ATCC 49138, and *Candida albicans* ATCC10231. Clinical isolates were previously collected and identified in our lab, including *E. faecalis* 32 (*optrA*^+^), *L. monocytogenes* LM08 (*tetM* ^+^), *E. coli* A3 (O157:H7), *E. coli* XG-E1 (*bla*_NDM–5_^+^ and *mcr-1*^+^), *E. coli* 47EC (*tet*(X4)^+^), *Klebsiella pneumoniae* XG-Kpn03 (*bla*_NDM–5_^+^), *Acinetobacter baumannii* 34AB (*tet*(X3)^+^), *Pseudomonas aeruginosa* 42 (*bla*_OXA–10_), *Pseudomonas fluorescens* AC04, and *Fusarium oxysporum* LD21. All bacterial strains were stored in 20% glycerol at −80°C. For the subsequent experiments, the bacteria were recovered and cultured in the corresponding enrichment broth at 37°C.

The MAC-T cell line and mouse macrophage leukemia cells RAW 264.7 were cultured in Dulbecco’s modified Eagle’s medium (DMEM), supplemented with 10% (v/v) of fetal bovine serum (Sigma-Aldrich, MO, United States), 100 U/mL of penicillin and 0.1 mg/mL of streptomycin, at 37°C in the presence of 5% CO_2_.

Female ICR mice (∼18 g, four weeks old) bought from Yangzhou University were used in this study. They were reared with *ad libitum* access to drinking water and antibiotic-free feed. The animal experiments were conducted in strict accordance with the Laboratory Animal Welfare and Animal Experimental Ethical Certificate, as issued by Committee on Animal Welfare and Experimental Ethics in Jiangsu Academy of Agricultural Sciences (approval no. JAAS20200516 and JAAS20211208).

### Antimicrobial Susceptibility Testing

To compare the antibacterial activity of RW-BP100 and its serial derivatives, the MIC against *S. aureus* ATCC29213 and *E. coli* ATCC25922 were determined using broth microdilution method according to the guidelines of Clinical and Laboratory Standards Institute (CLSI) M100-S25 (CLSI, 2015). Briefly, peptides were dissolved in sterilized water and twofold diluted in Mueller-Hinton (MH) broth (Beijing Land Bridge Technology) at the concentration ranges of 0.098–100 μg/mL. Then the peptides were mixed with an equal volume (100 μL) of bacterial suspensions of 10^6^ CFU/mL in a sterilized 96-well microtiter plate. After incubation at 37°C for 18 h, the MIC values were determined as the lowest concentrations of peptides with no visible growth of bacteria. To further evaluate the antimicrobial spectrum of RW-BP100-4D, MICs were determined against a more variety of Gram-positive and Gram-negative bacterial species, and the fungal strains (according to CLSI M38 guideline). At the end of incubation, aliquots (100 μL) of the bacterial solutions were placed on MH agar plates, and allowed growth at 37°C overnight for measurement of the minimal bactericidal concentration (MBC). The lowest peptide concentration that displayed no bacterial growth (zero colony) was defined as MBC. MIC and MBC testing were performed with three independent repeats.

### Hemolytic and Cytotoxicity Assays

To screen the peptide with lower toxicity, the hemolytic activity of RW-BP100 and its derivatives was tested using fresh defibrinated sheep blood (Beijing Land Bridge Technology company) (Song et al., 2020). Firstly, the sheep blood was centrifuged at 6,000 *g* for 5 min, washed with phosphate-buffered saline (PBS, 0.01 M, pH 7.4) for three times, and then diluted to the 8% (v/v) red blood cell suspension in PBS. All the peptides were dissolved in PBS and diluted to the concentrations of 100, 200, 300, and 600 μg/mL, respectively. Aliquots (100 μL) of the peptides were mixed with an equal volume of the 8% red blood cell suspension and incubated at 37°C for 1 h. The mixture was then centrifuged at 3,500 *g* for 10 min and 100 μL of the supernatant was transferred to a 96-well microplate to detect the OD576nm using Infinite M200 Microplate reader (Tecan, Switzerland). Melittin and PBS were used as positive and negative controls. The percentage of hemolysis (H) was calculated as follows: H = 100% × [(O_p_-O_b_)/(O_m_-O_b_)], where O_p_, O_b_, and O_m_ represented the densities of a given peptide, PBS buffer and melittin, respectively. Each test was performed with three independent repeats.

The cytotoxicity of RW-BP100 and its derivatives toward MAC-T and RAW264.7 cells was determined using the water-soluble tetrazolium salt-1 (WST-1) viability assay (Kai et al., 2011). Briefly, the MAC-T and RAW264.7 cells (10^3^ cells/mL) were seeded in a 96-well plate and then added with different peptide (150 μg/mL) for 24 h, respectively. Next, 10 μL of WST-1 (Beyotime, China) was added to each well to incubate for 1 h. The absorbance of the suspensions at 450 nm was measured as described above. Moreover, RW-BP100-4D was determined for its cytotoxicity toward MAC-T and RAW264.7 cells at the concentration ranges of 0–512 μg/mL. The cell survival rate was calculated as follows: (OD_drug_ – OD_blank_)/(OD_control_ – OD_*Blank*_) × 100%, where OD_drug_, OD_blank_, and OD_control_ are the densities of cultured cells treated with different concentrations of peptide, the culture medium, and growing cells without any treatment, respectively. Each test was conducted with three independent repeats.

### Stress Stability Testing

To evaluate the thermal and pH stability of RW-BP100-4D, 100 μL of the peptide (100 μg/mL, in PBS buffer) was incubated under different temperatures (40, 50, 60, 70, 80, 90, and 100°C, pH 7) or different pHs (2, 3, 4, 5, 6, 7, 8 and 9, 37°C) for 1 h (Song et al., 2020). After incubation, the pH of the peptide solutions was adjusted to 7.0 using NaOH or HCl. For protease and serum stability test, 100 μL of RW-BP100-4D (100 μg/mL) was incubated with proteinase K (1 mg/mL), trypsin (1 mg/mL), papain (1 mg/mL), or sterile calf serum at 37°C for 1 h, respectively (Song et al., 2020). After the treatments, the proteases and calf serum were inactivated by heating at 80°C for 30 min. The remnant activity of the treated RW-BP100-4D was evaluated by determining the MICs against *E. coli* ATCC25922, *S. aureus* ATCC29213, and *C. albicans* ATCC10231 using broth microdilution method. RW-BP100-4D without any treatment was used as the control and each test was conducted with three independent repeats. Moreover, degradation rate (%) was estimated as the percentage of degraded peptide calculated from the decrease of the high-performance liquid chromatography (HPLC) peak area of the native peptide. HPLC analysis was performed using the Agilent™ 1100 Series equipped with an Agilent analysis column (ZORBAX RX-C8, 2.1 mm × 150 mm, 5 μm) and an UV detector at 220 nm. Mobile phase was composed of A (H_2_O) and B (acetonitrile), which contained 0.065% trifluoroacetic (v/v) and 0.05% trifluoroacetic (v/v), respectively.

### *In vitro* Anti-biofilm Activity

The activity of RW-BP100-4D against biofilm-formation and mature biofilms in *S. aureus* ATCC29213 was tested as described previously (Gakhar et al., 2010). For biofilm-formation inhibition assay, aliquots (100 μL) of the bacterial cultures (5 × 10^5^CFU/mL) were added to a 96-well plate and incubated with 100 μL of different concentrations (0.78-100 μg/mL) of RW-BP100-4D or RW-BP100 for 24 h at 37°C, respectively. For the mature biofilms, 100 μL of the bacterial solutions (5 × 10^5^CFU/mL) were cultured in a sterile 96-well plate for 24 h. RW-BP100-4D or RW-BP100 at different concentrations (0.78–200 μg/mL) were added, respectively and incubated at 37°C for 30 min. After different treatments, all the wells were washed with sterilized PBS for three times, fixed with 200 μL of anhydrous methanol for 15 min, and then stained with 150 μL crystal violet (0.1%) for 15 min. The plates were rinsed in water for five times and air dried in room temperature. Lastly, the stained biofilms were completely dissolved in 33% ethanoic acid (100 μL) and the sample absorbance was measured at OD590 nm. Each test was performed with three independent repeats.

### Intracellular Antibacterial Activity

Confocal fluorescence assay was performed to investigate the uptaken ability of RW-BP100-4D by mammalian cells. MAC-T cells (10^4^ cells/mL) were cultured in glass-based dishes (Thermo Fisher Scientific) for 24 h, then N-terminal fluorescein isothiocyanate (FITC)-labeled RW-BP100-4D was added at a final concentration of 50 μg/mL and incubated for 2 h. After three times washing with PBS, the cells were fixed with cold methanol for 30 min, and stained with phalloidin and 6-diamidino-2-phenylindole (DAPI) for 30 and 5 min, sequentially. Fluorescence was observed using a Zeiss confocal fluorescence microscope (LSM 800, Carl Zeiss, Oberkochen, Germany). Fluorescence intensity was analyzed with Image J version 1.8.0.

The intracellular antibacterial activity of RW-BP100-4D against *S. aureus* ATCC29213 and *S. enterica* ATCC13076 were determined as described previously (John et al., 2019). MAC-T cells were cultured in a 12-well plate and then infected with *S. aureus* ATCC29213 or *S. enterica* ATCC13076 (∼10^5^CFU, MOI = 10) for 1 h. The spare bacteria were eliminated by treating with kanamycin (100 μg/mL) for 1 h. After that, kanamycin was removed by washing with PBS for 4 times. Then RW-BP100-4D or RW-BP100 were added to a final concentration of 50 μg/mL, respectively and incubated for 2 h. Meanwhile, kanamycin (50 μg/mL) and PBS were set as the antibiotic treatment group and treatment control group, respectively. Finally, MAC-T cells were treated with 0.1% TritonX-100. The lysates were serially diluted and spread on selective agar plates to enumerate the intracellular live bacteria. Each test was performed with three independent repeats.

### Effect on Chicken Meat Disinfection

The raw chicken meat was cut to 1 cm^3^ square pieces (about 0.25 g), then disinfected by washing with sodium hypochlorite, 75% ethanol, sterilized PBS for three times, and UV radiation for 1 h, sequentially. Bacterial strains of *S*. *aureus* ATCC29213, *L*. *monocytogenes* ATCC19115, *E. coli* O157:H7 clinical strain A3, *S. enterica* ATCC13076, *C. jejuni* ATCC 33291, and *S. putrefaction* ATCC 49138 (∼5 × 10^3^ CFU) were evenly spread on the surface of the chicken meat squares, individually or mixed. After bacterial contamination at 4°C for 1 h, the meat pieces were soaked in 1 mL of RW-BP100-4D (50 μg/mL) at 4°C for 24 h. PBS was used as the negative control. Lastly, the chicken pieces were homogenized and centrifuged, then the supernatants were diluted and spread on the corresponding selective medium to enumerate the residual live bacteria.

### Treatment Efficacy in Animal Models

The mouse skin infection model and bacteremia model were established to evaluate the treatment efficacy of RW-BP100-4D on local and system infections, respectively. In each model, ICR female mice were equally allocated into different treatment groups and each group contained six mice. The mouse skin infection model and bacteremia model were constructed according to previously described methods (Natalia et al., 2019; Song et al., 2020). The mice were challenged with methicillin-resistant *S. aureus* ATCC33591 (*mecA*+, MRSA) *via* spreading on skin lesion (5 × 10^5^ CFU/mL, 0.1 mL) or intraperitoneal injection (3.0 × 10^8^CFU/mL, 0.5 mL). After infection for 2 h, RW-BP100-4D or RW-BP100 were administrated to the local skin lesion of each mouse at 50 μg (1 mg/mL, 50 μL) for skin infection model, respectively. Ampicillin (1 mg/mL, 100 μL) and PBS were used as the antibiotic treatment control and negative control, respectively. In bacteremia model, peptides (RW-BP100-4D or RW-BP100) were injected intraperitoneally after 2 h of infection at different dose for each mice group (25, 50, and 100 mg/kg, respectively). Ampicillin (100 mg/kg) and PBS were used as the antibiotic treatment control and negative control, respectively. After treatment for 24 h, all the mice were sacrificed to collect the infected wound skin and infected organs (heart, liver, and kidney) from each model. *S. aureus* isolates were counted by using the Chromagar selective agar (Chromagar, France) supplemented with 8 μg/mL oxacillin.

### Statistical Analyses

Statistical analysis was performed using GraphPad Prism software (Version 8.2.1). All data were presented as the mean ± S.D. *p* values were calculated using an independent two sample *t*-test for the difference in CFU after different treatments. *p*-values <0.05 (*) were considered statistically significant.

## Results

### Antimicrobial Activity

Minimal inhibitory concentration was determined to evaluate the antimicrobial activity of RW-BP100 and its derivatives (Table 1). *S. aureus* ATCC29213 and *E. coli* ATCC25922 were chosen as the representative Gram-positive and Gram-negative bacteria, respectively. The results indicated that the MICs of the parental peptide RW-BP100 against *S. aureus* ATCC29213 and *E. coli* ATCC25922 were both 12 μg/mL, whereas the derivatives RW-BP100-4D, RW-BP100-5D, RW-BP100-6D, RW-BP100-8D, and RW-BP100-All-D displayed equal or better antibacterial activities (MIC = 3–12 μg/mL). In contrast, the transformation of N terminal (9th–11th) amino acids into corresponding D-enantiomers (RW-BP100-9D, RW-BP100-10D, and RW-BP100-11D) decreased the antimicrobial activity, especially that of RW-BP100-11D (MIC > 50 μg/mL). These results indicated that the 11th amino acid was crucial for the activity of RW-BP100 and could not be replaced.

Among these derivatives, RW-BP100-4D showed the best antimicrobial activity, with the MIC of 3 μg/mL against *S. aureus* ATCC29213 and 6 μg/mL against *E. coli* ATCC25922. Subsequently, the antibacterial spectrum of RW-BP100-4D was determined against a more variety of microorganisms compared to that of RW-BP100. As shown in Table 2, the MICs of RW-BP100-4D ranged from 3 to 12 μg/mL against the selected Gram-positive bacterial isolates (*S. aureus, S. epidermidis, E. faecalis, S. agalactiae*, and *L. monocytogenes*), and 3–25 μg/mL against the selected Gram-negative bacterial isolates (*E. coli, Salmonella enterica, K. pneumoniae, C. jejuni, A. baumannii, Pseudomonas* spp., and *S. putrefaction*), respectively. Besides, RW-BP100-4D was active toward fungal isolates and the MICs against *C. albicans* and *F. oxysporum* were both 12.5 μg/mL. Compared to RW-BP100, RW-BP100-4D retained the antibacterial activity against Gram-negative bacteria and fungi, and improved its activity against some Gram-positive bacteria, including both the sensitive and resistant strains. The MBC values of RW-BP100-4D were usually two fold higher than the corresponding MIC values, whereas the MBC values of RW-BP100 were 4–8 fold of the corresponding MIC values, indicating that RW-BP100-4D possessed better antimicrobial activity than its parental peptide RW-BP100, and may display a bactericidal action towards the target pathogens.

**TABLE 2:** Antimicrobial activity of RW-BP100 and RW-BP100-4D against various microorganisms.

	Antimicrobial resistance genes	MIC of peptides (μg/mL)		MBC of peptides (μg/mL)	
		RW-BP100	RW-BP100-4D	RW-BP100	RW-BP100-4D
Gram-positive bacteria					
*Staphylococcus aureus* ATCC29213	ND^a^	6^b^	3	50	6
*Staphylococcus aureus* ATCC33591	*mec*A	6	3	50	6
*Staphylococcus epidermidis* ATCC29887	ND	3	3	12	6
*Enterococcus faecalis* JH2-2	ND	25	12	50	50
*Enterococcus faecalis* 32	*optr*A	25	12	50	50
*Streptococcus agalactiae* ATCC13813	ND	3	3	12	6
*Listeria monocytogenes* ATCC19115	ND	6	3	50	6
*Listeria monocytogenes* LM08	*tetM*	6	3	50	6
Gram-negative bacteria					
*Escherichia coli* ACTT25922	ND	6	6	25	12
*Escherichia coli* O157:H7	ND	3	3	25	6
*Escherichia coli* XG-E1	*bla*_NDM–5_, *mcr-1*	3	3	25	6
*Escherichia coli* 47EC	*tet*(X4)	6	6	25	12
*Salmonella enterica* ATCC13076	ND	6	3	25	6
*Klebsiella pneumoniae* XG-Kpn03	*bla*_NDM–5_	25	25	50	50
*Campylobacter jejuni* ATCC33291	ND	6	6	25	12
*Acinetobacter baumannii* 34AB	*tet*(X3)	6	6	25	12
*Pseudomonas aeruginosa* 42	ND	12	12	50	50
*Pseudomonas fluorescens* AC04	ND	6	6	25	12
*Shewanella putrefaction* ATCC49138	ND	6	6	25	12
Fungi					
*Candida albicans* ATCC10231	ND	12	12	50	25
*Fusarium oxysporum* LD21	ND	12	12	50	25

*^a^ND means not detected.*

*^b^MIC and MBC values were the mean of three repeated experiments.*

### Hemolytic Activity and Cytotoxicity

As shown in Table 1, the hemolytic rates of RW-BP100 to sheep blood cells were 3.10 and 4.70% at the concentration of 50 and 100 μg/mL, respectively, which significantly increased to 41.82 and 56.67% at the peptide concentrations of 150 and 300 μg/mL, respectively. The derivatives exhibited low hemolytic rates (0.11–5.99%) at 50–100 μg/mL; however, the hemolytic rates increased substantially (16.17–41.82%) at 150 μg/mL and most of the peptides have hemolysis of >40% at 300 μg/mL. A comparison of hemolytic rates between RW-BP100 and its derivatives at 150–300 μg/mL revealed that both single D-amino acid substitution and All-D-isomers led to reduced hemolytic activity. Especially, single D-amino acid substitution at the 4th, 7th, 9th, 10th, or 11th positions deceased the hemolytic rate to <20% at 150 μg/mL. For cytotoxicity assay, the survival rates of MAC-T and RAW264.7 cells were 57.80 and 36.29%, respectively, when treated with RW-BP100 at 150 μg/mL (Table 1). In contrast, single D-amino acid substitution (4th, 7th, 9th or 10th positions) largely increased the survival rates to >90 and >70% for MAC-T and RAW264.7 cells, respectively (Table 1). Among these derivatives, RW-BP100-7D showed the lowest cytotoxicity, with the survival rates of MAC-T and RAW264.7 cells (at peptide concentration of 150 μg/mL) reaching 96.41 and 93.74%, respectively. However, the antimicrobial activity of RW-BP100-7D was much weaker than that of RW-BP100-4D, which also possessed relatively lower cytotoxicity among these derivatives (Tables 1, 2 and Supplementary Figure 1). Taken together, RW-BP100-4D was selected for further stability and activity evaluation.

### Thermal, pH, Protease, and Serum Stability

The stability of RW-BP100-4D was evaluated under different temperature, pH, and protease condition (Supplementary Table 1). After incubation at temperatures from 40 to 80°C, the MICs of RW-BP100-4D against *E. coli* ATCC25922, *S. aureus* ATCC29213, and *C. albicans* ATCC10231 remained unchanged (6, 3 and 12 μg/mL, respectively) in all three repeats. However, incubation at 90 and 100°C caused 2–8 fold increase of RW-BP100-4D MICs against the corresponding reference strains. HPLC determination revealed that 56 and 82% of the original peptide were disrupted at 90 and 100°C, respectively (Supplementary Table 1). The results indicated that RW-BP100-4D maintained its activity at the temperature below 80°C, which is consistent with the previous study showing that AMPs were more tolerant to high temperatures (Tanhaeian et al., 2020). With respect to pH sensitivity, the MICs of RW-BP100-4D against the three reference strains remained stable at the pH ranges of 2–7, but increased 2–4 fold at the pHs of 8 and 9. This result indicated that RW-BP100-4D was more tolerant to the acidic environment than alkaline conditions, which might be due to the disruption of peptide bonds at higher pHs (Jones et al., 2015). Treatment with proteinase K increased the MICs of RW-BP100-4D against the corresponding strains to 4–8 fold of the original level. Likewise, treatment with papain, trypsin, or calf serum significantly reduced the antimicrobial activity (MIC > 50 μg/mL and >95% degradation). These results indicated that RW-BP100-4D was vulnerable to proteases and may not be stable in the serum.

### Anti-biofilm Activity *in vitro*

**FIGURE 2:**
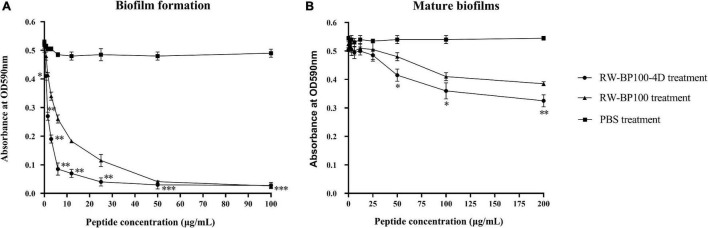
Anti-biofilm activity of RW-BP100-4D against *S. aureus* ATCC29213. **(A)** Activity of RW-BP100-4D against the biofilm-formation of *S. aureus* ATCC29213 **(B)** Activity of RW-BP100-4D against the mature biofilm of *S. aureus* ATCC29213. Data are the mean ± SD of triplicate wells from three independent experiments. *p* values were calculated using an independent two sample *t*-test for the difference in absorbance at OD590nm between RW-BP100-4D-treated group and the PBS control, *p < 0.5, ^**^p < 0.01, ^***^p < 0.001.

As shown in Figure 2A, RW-BP100-4D effectively inhibited the biofilm formation of *S. aureus* ATCC29213 and the inhibition ability was significantly enhanced as the concentration of RW-BP100-4D increased from 0.78 to 100 μg/mL compared to the PBS control (*p* < 0.05). In contrast, RW-BP100 also inhibited the biofilm formation of *S. aureus* ATCC29213 and the inhibition ability of RW-BP100 was weaker than that of RW-BP100-4D (Figure 2A). Compared with the PBS control, 82.3 and 45.8% of the biofilm in *S. aureus* was inhibited by RW-BP100-4D and RW-BP100 at 6.25 μg/mL, respectively. Although the anti-biofilm activity was concentration-dependent, the activity was slowly increased when RW-BP100-4D and RW-BP100 concentrations were above 12.5 and 50 μg/mL, respectively, which may be due to that the peptides at concentrations near the MBC of the target strain killed most of the bacteria. For mature biofilms, treatment with lower concentrations (≤4 × MIC) of RW-BP100-4D led to a slight loss in biomass (Figure 2B). However, higher concentrations (50–200 μg/mL) slightly improved the anti-biofilm activity and about 40.4% of the biofilm matrix was lost after treatment with 200 μg/mL of RW-BP100-4D compared to the PBS control (*p* < 0.01). In contrast, RW-BP100 showed a weaker anti-biofilm activity than that of RW-BP100-4D and 29.4% of the biofilm matrix was lost after treatment with 200 μg/mL of RW-BP100.

### Antibacterial Activity in the MAC-T Cells

**FIGURE 3:**
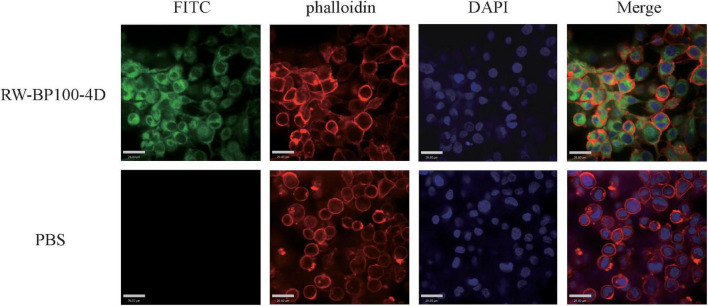
Confocal fluorescence images showing distribution of RW-BP100-4D in MAC-T cells. The up panel indicates the RW-BP100-4D treated group. The down panel indicates the PBS treated control group. Green color indicates FITC-labeled peptide RW-BP100-4D, red indicates the phalloidin-stained MAC-T cell membrane and blue indicates the DAPI-stained cell nucleus. The scale bar is 29.00 μm. FITC, Fluorescein isothiocyanate; DAPI, 6-diamidino-2-phenylindole.

**FIGURE 4:**
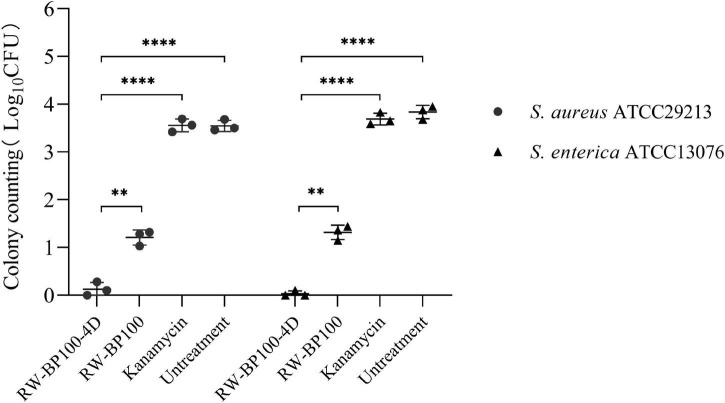
Intracellular antibacterial activities of RW-BP100-4D against *S. aureus* ATCC29213 and *S. enterica* ATCC13076 within the MAC-T cells. Kanamycin and PBS were used as the antibiotic-treatment and untreated controls, respectively. Data are the mean ± SD of triplicates from three independent experiments. *p* values were calculated using an independent two sample *t*-test for the difference in CFU after different treatments, ^**^p < 0.01, ^****^p < 0.0001.

The confocal fluorescence images in Figure 3 showed that the FITC-labeled RW-BP100-4D at 50 μg/mL was rapidly (≤2 h) and efficiently uptaken by the MAC-T cells and evenly distributed throughout the whole cytoplasm. To further investigate the intracellular antibacterial activity of RW-BP100-4D, *S. aureus* ATCC29213 and *S. enterica* ATCC13076 were used as the target pathogens. It showed that both *S. aureus* and *S. enterica* at an initial dose of 5 × 10^5^ CFU could infect and colonize in the MAC-T cells at CFU of 3.55 × 10^3^ and 6.92 × 10^3^, respectively (Figure 4). Notably, RW-BP100-4D at 50 μg/mL could significantly eliminate the intracellular *S. aureus* ATCC29213 by 3.42 log and *S. enterica* ATCC13076 by 3.81 log, compared with that of kanamycin treatments (*p* < 0.0001). In contrast, RW-BP100 at 50 μg/mL killed the intracellular *S. aureus* ATCC29213 by 2.34 log and *S. enterica* ATCC13076 by 2.57 log. RW-BP100-4D showed better intracellular antibacterial activity than that of RW-BP100 (*p* < 0.01) and there was no significant difference between the CFU of kanamycin treatment group and the untreated group (Figure 4).

### Disinfection Effect of RW-BP100-4D on Chicken Meat

**FIGURE 5:**
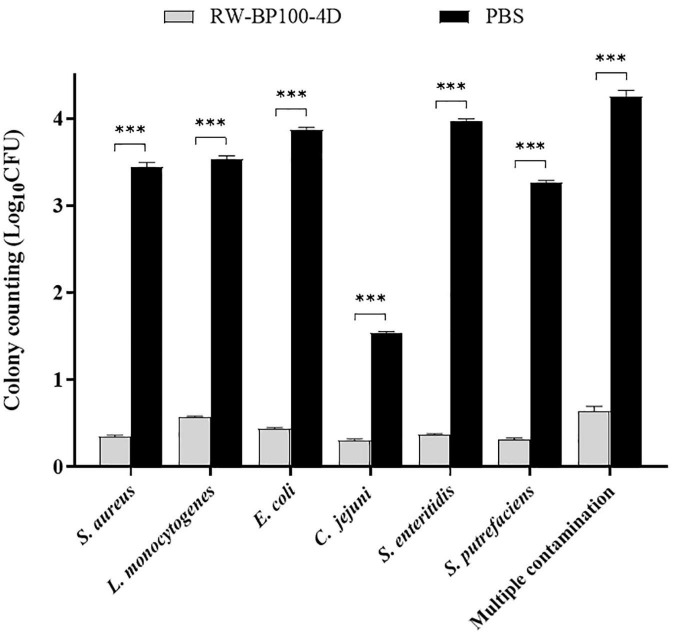
Effect of RW-BP100-4D on chicken meat disinfection contaminated with various bacterial species. Multiple contamination indicates the chicken meat concurrently contaminated by *S. aureus*, *L. monocytogenes*, *E. coli* O157: H7, *C. jejuni*, *S. enterica*, and *S. putrefaciens*. Data are the mean ± SD from three independent experiments. Independent two sample *t*-test was used to compare the difference of CFU between RW-BP100-4D-treatment group and PBS control group, ^***^p < 0.001.

The potential application of RW-BP100-4D to disinfect food was tested by using chicken meat manually contaminated by diverse bacteria. As shown in Figure 5, RW-BP100-4D effectively reduced the bacterial loads on chicken meat, including *S. aureus* by 3.09 ± 0.035 log (99.92% killing), *L. monocytogenes* by 2.96 ± 0.028 log (99.89% killing), *E. coli* O157: H7 by 3.43 ± 0.021 log (99.96% killing), *C. jejuni* by 1.24 ± 0.017 log (94.18% killing), *S. enteritidis* by 3.60 ± 0.021 log (99.97% killing) and *S. putrefaciens* by 2.95 ± 0.024 log (99.89% killing), respectively. There were significant differences between the CFU of RW-BP100-4D treatment group and the corresponding controls (*p* < 0.001) for each bacterial strain. Interestingly, RW-BP100-4D could also reduce the bacterial loads of multiple contamination (concurrently contaminated by *S. aureus*, *L. monocytogenes*, *E. coli* O157: H7, *C. jejuni*, *S. enterica*, and *S. putrefaciens*) by 3.61 ± 0.063 log in total, indicating the potential effectiveness of RW-BP100-4D in food disinfection.

### Treatment Efficacy in Mouse Skin Infection and Bacteremia Models

**FIGURE 6:**
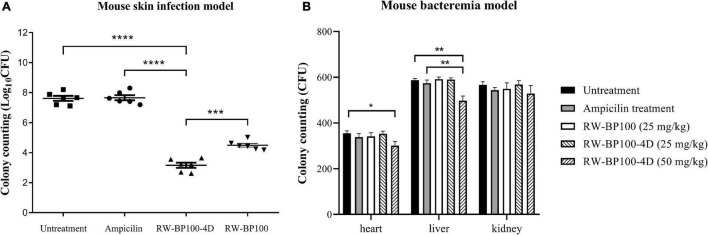
Treatment efficacy of RW-BP100-4D against *S. aureus* ATCC33591 in **(A)** mouse skin infection model and **(B)** mouse bacteremia model. Ampicilin and PBS were used as the antibiotic-treatment and untreatment controls, respectively. Data are the mean ± SD from three independent experiments. Independent two sample *t*-test was used to compare the difference of CFU between different treatment groups, *p < 0.5, **p < 0.01, ***p < 0.001, ****p < 0.0001.

The *mecA*-positive *S. aureus* (MRSA) ATCC33591 was used to establish the mouse skin infection model. In the control group, the bacterial load in the infected mice skin reached 4.12 × 10^7^ CFU after 24 h (Figure 6A). RW-BP100-4D reduced *S. aureus* ATCC33591 by >4 log orders of magnitude (99.99% killing) over a 24 h period, while ampicillin did not decrease the bacteria load. Statistical analysis revealed that there was a significant difference of CFU in the infection sites between RW-BP100-4D treatment group and ampicillin treatment group (*p* < 0.0001), whereas no significant difference was found between the ampicillin treatment group and the untreatment controls. Although RW-BP100 also showed good efficacy in reducing bacterial load (3.13 log reduction) in the infected mice skin, there was a significant difference between CFU of RW-BP100-4D and RW-BP100 treatment group (*p* < 0.001, Figure 6A). For bacteremia model, the bacterial loads in the mouse’s heart, liver and kidney in the control group reached 3.55 × 10^2^, 5.87 × 10^2^ and 5.67 × 10^2^ CFU, respectively. RW-BP100-4D administrated at 100 mg/kg and RW-BP100 at 50 or 100 mg/kg caused the death of >80% mice. Therefore, the bacterial loads were only counted in mice groups that were treated with the other concentrations. It revealed that RW-BP100 or RW-BP100-4D administrated at 25 mg/kg did not significantly reduce the bacterial loads in these organs. However, RW-BP100-4D administrated at 50 mg/kg significantly reduced the bacterial loads in the mouse’s heart and liver (*p* < 0.5), indicating a modest treatment efficacy of RW-BP100-4D in mouse systemic infections (Figure 6B).

## Discussion

In this study, we designed a series of derivatives from RW-BP100 and screened out the ideal candidate RW-BP100-4D, which showed relatively low cytotoxicity and the best antimicrobial activity. Generally, the activity of AMP is infuenced by many factors, such as peptide length, net charge, hydrophobicity and secondary structure (Zhang et al., 2021). Since single D-amino acid transformation does not alter the length and net charge of the parental peptide, the enhanced antimicrobial activity of RW-BP100-4D in this study may be largely due to the variation of peptide hydrophobicity or secondary structure, thus leading to a stronger binding affinity with the anionic components on the microbial membrane, such as LPS of Gram-negative bacteria, lipoteichoic acid of Gram-positive bacteria and mannan of fungi (Zhang et al., 2021). Notably, the antimicrobial activity of RW-BP100-4D against antibiotic-susceptible and resistant strains were very similar, such as methicillin-sensitive *S. aureus* ATCC29213 versus methicillin-resistant *S. aureus* ATCC33591 (both MICs were 6 μg/mL), and antibiotic-sensitive *E.coli* ATCC25922 (MIC = 6 μg/mL) versus tigecycline-resistant *E. coli* 47EC (MIC = 3 μg/mL), indicating that the antimicrobial activity of RW-BP100-4D was indiscriminately and not affected by the resistance genes carried by the bacteria, which may be due to the special bactericidal mechanisms of the peptide (Toke, 2005).

From the hemolytic activity and cytotoxicity assays, we could see that the derivatives with single D-amino acid substitution at the 4th, 7th, 9th, 10th or 11th positions showed decreased cytotoxicity compared to the parental peptide RW-BP100. These results were consistent with the previous study, which showed that subtle changes in a peptide influenced not only the antimicrobial activity but also the hemolytic activity (Imma et al., 2011). Other studies have reported excellent *in vitro* antimicrobial activity profiles of several D-enantiomer peptides. However, the high production costs imposed severe limitations to their application as antimicrobial products (McGrath et al., 2013; Chen et al., 2016). Considering the short length of only 11 amino acids and just one D-amino acid replacement, which could largely reduce the cost of peptide synthesis, RW-BP100-4D can be taken as a promising candidate for further development in antimicrobial application.

As more than 95% of the bacteria in nature are existing in biofilms, which could help them resistant to antibiotics and the host immune system, and played an important role in the persistence of chronic infections (Imma et al., 2011; Kharidia and Liang, 2011), it is of great significance to eradicate the bacterial biofilms in the clinical treatment. Findings in this study indicated that RW-BP100-4D showed better anti-biofilm activity *in vitro* than its parental peptide RW-BP100. RW-BP100-4D showed a dose-dependent anti-biofilm activity and the biofilm-formation in *S. aureus* was completely inhibited when peptide concentrations were kept at a lower concentration (≥1 × MIC). It is well known that mature biofilms are more common and complicated in human clinical treatment, especially the implants associated infections (Costerton et al., 2007). Interestingly, RW-BP100-4D also showed modest dispersal activity against mature biofilms of *S. aureus* when applied with a higher dose (>8 × MIC). Despite RW-BP100-4D displayed good biofilm-inhibition ability *in vitro* against *S. aureus*, further studies are still needed to prove its *in vivo* anti-biofilm activity as some AMPs may have their activity nullified in the presence of tryptone, proteins, or high concentration of salts in the body fluid (Beckloff et al., 2007).

Previous studies have shown that some short cationic peptides could be used as cell-penetrating carrier to transport functional cargoes (such as DNA, proteins, and chemical agents) into the target cells (Mazel et al., 2001; Perea et al., 2004). As RW-BP100-4D has an amphiphilic structure that may easily penetrate through the hydrophobic lipid bilayer, this short peptide is tested for its cell-penetrating ability. The results revealed that RW-BP100-4D could efficiently penetrate into the MAC-T cells and concurrently kill the *S. aureus* and *S. enterica* isolates colonized within the cells. Intracellular bacteria, including opportunistic pathogens (such as *S. aureus* and *Salmonella* spp.) and obligate parasites (e.g., *mycobacterium tuberculosis* and *Brucella* spp.), posed a great challenge to clinical treatment since these microorganisms could colonize within the cells and thus evade the killing of some clinically-important antibiotics such as β-lactams and aminoglycosides (Paul and AndrÉ, 1978; Petra and Matthias, 2005). Although RW-BP100 also showed good intracellular bacterial-killing ability, the cytotoxic concentration of RW-BP100 was much higher than that of RW-BP100-4D. Therefore, the modified peptide has a higher therapeutic index and may be more appropriate to be developed in treating the intracellular bacterial infections or as a cell-penetrating tool to deliver the functional molecules in the future.

*Staphylococcus aureus*, *L. monocytogenes*, *E. coli* O157: H7, *C. jejuni*, and *Salmonella* spp. were the main foodborne pathogens commonly identified in food poisoning incidences (Kirk et al., 2015), and *S. putrefaciens* was one of the dominated pathogens that led to food spoilage of chilled meat (Lan et al., 2019). The present study showed that RW-BP100-4D was active against the above-mentioned pathogens contaminated on chicken meat, either by single microorganism or by multiple pathogens. Previous study has reported that Nisin, which is a natural antimicrobial peptide and has been commercialized as a food preservative, is active against many foodborne pathogens such as *L. monocytogenes* and *Clostridium botulinumon* (Gharsallaoui et al., 2016). However, the antimicrobial spectrum of Nisin is limited to the Gram-positive bacteria, and Gram-negative bacteria are usually resistant to nisin mainly due to their impermeable outer membranes (Gharsallaoui et al., 2016). Therefore, RW-BP100-4D may have the advantages in food disinfection as it could reduce the transmission risk of foodborne pathogens, including both Gram-postitive and Gram-negative bacteria. In addition to food disinfection, RW-BP100-4D also displayed good efficacy in treating MRSA infection in mouse local skin. However, the *in vivo* treatment efficacy of RW-BP100-4D toward MRSA was modest in mouse bacteremia model, which may be due to its degradation by proteolytic enzymes in the animal serum or tissues. Moreover, a higher dose (100 mg/kg) of RW-BP100-4D led to the death of most treated mice, indicating that the candidate peptide is toxic to the animals when applied *in vivo* with higher doses. Development of AMP-based application has encountered many challenges including stability, efficacy and toxicity (Zhang et al., 2021). As indicated in this study, RW-BP100-4D showed generally good bactericidal effect *in vitro*, including the removal of food bacteria and treatment of local skin infection, which has a potential to be applied as a food bactericide and local external use. However, the toxicity of RW-BP100-4D should not be ignored (hemolysis at higher concentrations) when the candidate peptide is to be developed on these applications. Moreover, both toxicity and protease-sensitivity of RW-BP100-4D are unavoidable problems when used *in vivo*, which will limit the development of the peptide in clinical therapy, including the biofilm removal and systemic infection treatment. Therefore, future research works are still needed to further reduce the toxicity and improve the therapeutic effect of RW-BP100-4D *in vivo*.

## Conclusion

This study presented a broad-spectrum AMP designated RW-BP100-4D, which was derived from RW-BP100 by transforming the N-terminal-4th amino acid from L- to D-enantiomer. Compared to its parent analogue, RW-BP100-4D showed remarkably reduced cytotoxicity and enhanced antimicrobial activity, including both Gram-positive and Gram-negative bacteria as well as the fungi. Moreover, RW-BP100-4D displayed good bactericidal effect in the removal of food bacteria and local skin infection. However, the protease-sensitivity of RW-BP100-4D and toxic effect at higher doses reduced the therapeutic effect of the candidate peptide *in vivo* and should be managed in the future studies.

## Data Availability Statement

The original contributions presented in the study are included in the article/Supplementary Material, further inquiries can be directed to the corresponding authors.

## Ethics Statement

The animal study was reviewed and approved by Committee on Animal Welfare and Experimental Ethics in Jiangsu Academy of Agricultural Sciences (approval no. JAAS20200516 and JAAS20211208).

## Author Contributions

RW and TH designed the study. TH, XT, JL, RCW, and LG conducted all the experiments. TH, JL, and XJ analyzed and interpreted the data. TH, XT, and JL wrote the manuscript. All authors reviewed, revised, and approved the final manuscript.

## Conflict of Interest

The authors declare that the research was conducted in the absence of any commercial or financial relationships that could be construed as a potential conflict of interest.

## Publisher’s Note

All claims expressed in this article are solely those of the authors and do not necessarily represent those of their affiliated organizations, or those of the publisher, the editors and the reviewers. Any product that may be evaluated in this article, or claim that may be made by its manufacturer, is not guaranteed or endorsed by the publisher.
