# Supplementary figures and images for: RW-BP100-4D, a Promising Antimicrobial Candidate With Broad-Spectrum Bactericidal Activity
Source: Front Microbiol. 2022 Jan 25;12:815980. doi: 10.3389/fmicb.2021.815980 (PMC8822125; doi:10.3389/fmicb.2021.815980)

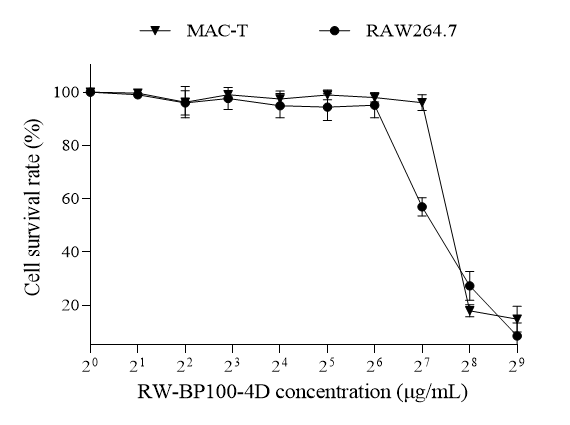

Supplement: Supplementary file 1 [file Image_1.TIF]
