# Supplementary material for: RW-BP100-4D, a Promising Antimicrobial Candidate With Broad-Spectrum Bactericidal Activity
Source: Front Microbiol. 2022 Jan 25;12:815980. doi: 10.3389/fmicb.2021.815980 (PMC8822125; doi:10.3389/fmicb.2021.815980)
Supplement: Supplementary file 2 [file Table_1.DOC]

**Supplementary table 1. Stability of RW-BP100-4D under different temperature, pH and protease condition.**

|  | MIC (µg/mL) of RW-BP100-4D against ^a^ | | | Degradation ^b^  (%) |
| --- | --- | --- | --- | --- |
|  | *E. coli* ATCC25922 | *S. aureus* ATCC29213 | *C. albicans* ATCC10231 |  |
| 40-80 ℃ | 6 | 3 | 12 | 0 |
| 90 ℃ | 12 | 6 | 25 | 56 |
| 100 ℃ | 25 | 25 | 50 | 82 |
| pH 2-7 | 6 | 3 | 12 | — |
| pH 8-9 | 12 | 12 | 25 | — |
| papain | >50 | >50 | >50 | >90 |
| trypsin | >50 | >50 | >50 | >90 |
| calf serum | >50 | >50 | >50 | >90 |

^a^ MIC values were the mean of three repeated experiments.

^b^ Degradation (%) was estimated as the percentage of degraded peptide calculated from the decrease of the HPLC peak area of the native peptide.
